# Fractional Refined Composite Multiscale Fuzzy Entropy of International Stock Indices

**DOI:** 10.3390/e21090914

**Published:** 2019-09-19

**Authors:** Zhiyong Wu, Wei Zhang

**Affiliations:** School of Mathematics and Physics, and the Key Laboratory of Advanced Perception and Intelligent Control of High-End Equipment, Ministry of Education, Anhui Polytechnic University, Wuhu 241000, China; wuzhiyong10@gmail.com

**Keywords:** multiscale fuzzy entropy, composite multiscale fuzzy entropy, refined composite multiscale fuzzy entropy, fractional refined composite multiscale fuzzy entropy, complexity

## Abstract

Fractional refined composite multiscale fuzzy entropy (FRCMFE), which aims to relieve the large fluctuation of fuzzy entropy (FuzzyEn) measure and significantly discriminate different short-term financial time series with noise, is proposed to quantify the complexity dynamics of the international stock indices in the paper. To comprehend the FRCMFE, the complexity analyses of Gaussian white noise with different signal lengths, the random logarithmic returns and volatility series of the international stock indices are comparatively performed with multiscale fuzzy entropy (MFE), composite multiscale fuzzy entropy (CMFE) and refined composite multiscale fuzzy entropy (RCMFE). The empirical results show that the FRCMFE measure outperforms the traditional methods to some extent.

## 1. Introduction

It is generally believed that the logarithmic returns and volatility (which means absolute-price logarithmic returns in this paper) of international stock indices often possess strong nonlinearity and nonstationarity [1,2,3,4,5]. Exploring statistical characteristics, predictability and modeling of financial variables (returns, price, volume, etc.) has been a key objective for their significant importance in theoretical research and wide application in financial fields, such as risk management, derivatives pricing, forecasting and modeling [1,5,6,7]. Of course, the primary question is to judge whether a financial signal is worth modeling, i.e., we should judge whether the time series is random walk or regular to some extent, and how about the structural dynamics. In addition, the complexity of a time series is a measure, which may be related to the unpredictability and the difficulties in predicting a signal. The larger complexity a time series has, more difficulties in predicting there is. As expected, an irregular series should be more complex than a regular one, i.e., a pure stochastic series should have larger complexity value than a regular one in single scale case [8], and, possessing the partial past history and related structure information, a time series with long-range correlations has larger complexity than a pure stochastic series in multiscale case [9]. Recently, abundant complexity methods and corresponding improved measures have been proposed, for example, entropy measures, Lyapunov exponents and fractal dimension [10,11,12,13,14,15,16], where entropy measures are the most favorite for their simplicity in understanding and convenience for program with computing software. Enormous revised nonlinear measures based on permutation entropy (PermEn), approximate entroy (AppEn), sample entropy (SampEn) and fuzzy entropy (FuzzyEn) are proposed to detect the complexity dynamics of physiological, traffic and financial time series which are typically short, and commonly contaminated by noise [8,11,12,13,17,18,19,20,21,22,23,24,25,26,27,28,29,30]. Where SampEn is the improved version of AppEn with excluding self-matching, and FuzzyEn is the improved version of SampEn measure with fuzzy membership function in the place of the Heaviside function [8]. Fractional sample entropy (FSE), which combining traditional sample entropy with fractional calculus, is also an improved version of sample entropy method, and it can sensitively explore fractional order dynamics and evolutionary information in a nonlinear system, and hence get more accurate understanding of the series [22]. The random logarithmic returns and volatility series of financial data are proved to possess different complexity degree [22]. Composite multiscale entropy measure is proposed to quantify complexity of short-term financial time series, and it shows the advantages in stability and reliability of results when compared with the conventional algorithms [11]. Refined composite multiscale permutation entropy (RCMPE) is proposed based on PermEn and refine, composite, multiscale technologies to overcome length dependence and hence achieve more stable estimations than MPE [25]. Fractional fuzzy entropy (FFE) is proposed based on FuzzyEn and fractional information to explore complexity behavior of financial dynamics [27]. Combining fuzzy entropy with multiscale, composite and refine technologies, refined composite multiscale fuzzy entropy (RCMFE) is proposed to detect localized defect of rolling element bearing [28], and it is first applied to explore the complexity dynamics of returns and volatility series of the international stock indices in the paper. Many classical entropies, distances, etc. are generalized by combining them with the concepts of fractional calculus. And the novel measures exhibit superior sensitivity to the characteristics exhibited by each distinct type of data by tuning the fractional order in practical applications [31,32,33].

In this work, inspired by the works [21,22,28,31,32,33], where improved measures are proposed by the combination of traditional measures with fractional calculus, composite technology, etc. and can obtain more sensitive and stable analysis results, combining RCMFE with fractional order information and refine, composite technology, a revised complexity measure—fractional refined composite multiscale fuzzy entropy (FRCMFE)—is proposed to study the complexity behaviors of the returns and volatility of international stock indices, which is expected to investigate complexity behavior of noisy signal sensitively and stably with relatively short length, which is representative nature of financial time series. Moreover, through the analyses of the Gaussian series with different lengths and real market indices, the empirical results confirm that the proposed FRCMFE is superior to traditional complexity measures to some extent.

The remainder of the manuscript is organized as follows. Section 2 introduces the FuzzyEn, RCMFE, and FRCMFE methods briefly. In Section 3, Gaussian white noise is used to evaluate the effectiveness of MFE, RCMFE and FRCMFE. Section 4 presents the entropy results of returns and volatility series of international stock indices, followed by a conclusion in Section 5.

## 2. Methodologies

### 2.1. Fuzzy Entropy

The fuzzy entropy (FuzzyEn) measure, which combines the concept of fuzzy sets and vectors’ similarity defined in AppEn, SampEn, is a novel complexity measure, where vectors’ similarity is defined by fuzzy similarity degree based on fuzzy membership functions and vectors’ shapes in the place of Heaviside function.

Given a time series $x=\{x_{i},\;i=1,2,\cdots ,T\}$, the FuzzyEn value can be calculated as follows [8,21]. Construct a *m*-dimensional vector sequence with length T−m+1 $\left\{X_{i}^{m},1\leq i\leq T-m+1\right\}$ by the well-known method proposed by Takens [34] and subtract the mean value as:

(1) $$X_{i}^{m}=\left\{x_{i},x_{i+1},\cdots ,x_{i+m-1}\right\}-\bar{x}\left(i\right)$$

where the parameters *m* is called the embedding dimension, $\bar{x}\left(i\right)$ is the mean value of the vector $\left\{x_{i},x_{i+1},\cdots ,x_{i+m-1}\right\}$ for baseline removal, i.e.,

(2) $$\bar{x}\left(i\right)=\frac{1}{m}\sum_{j=0}^{m-1}x_{i+j}.$$

Every phase point of m-dimensional phase space $\left\{X_{i}^{m}\right\}$ represents a certainly instantaneous state of a system. Then, given vector series $\left\{X_{i}^{m}\right\}$, the similarity degree $D_{ij}^{m}$ of $X_{i}^{m}$ to its neighboring vector $X_{j}^{m}$ defined by a fuzzy membership function as:

(3) $$D_{ij}^{m}=e^{-{\left(d_{ij}^{m}/r\right)}^{n}}$$

where the parameters *n* is the gradient of boundary, *r* is the width of the fuzzy function, $d_{ij}$ is the maximum norm of difference vector of $X_{i}^{m}$ and $X_{j}^{m}$ in this paper. For all vectors $\left\{X_{i}^{m},1\leq i\leq T-m+1\right\}$, we can get the probability $C^{m}\left(r\right)$ by the mean values of $D_{ij}^{m}$ of any two vectors as:

(4) $$C^{m}\left(r\right)=\frac{1}{T-m}\sum_{i=1}^{T-m}\left(\frac{1}{T-m-1}\sum_{j=1,j\neq i}^{T-m}D_{ij}^{m}\right).$$

Obviously, $C^{m}\left(r\right)$ can represent similarity probability of any two vectors in the mean sense. Similarly, there also exists the probability $C^{m+1}\left(r\right)$ for m+1 dimension vectors series $\left\{X_{i}^{m+1},1\leq i\leq T-m\right\}$ as:

(5) $$C^{m+1}\left(r\right)=\frac{1}{T-m}\sum_{i=1}^{T-m}\left(\frac{1}{T-m-1}\sum_{j=1,j\neq i}^{T-m}D_{ij}^{m+1}\right).$$

Finally, for the time series *x*, the FuzzyEn is estimated as follows:

(6) $$\mathrm{FuzzyEn}\left(m,n,r,T\right)=-\ln\frac{C^{m+1}\left(r\right)}{C^{m}\left(r\right)}.$$

Generally, *m* and *n* (m,n>1) are set to two small values to avoid the loss of the detailed information, *n* is set to be 2 in this paper, and *r* should be multiplied by the standard deviation (SD) of the original dataset to avoid the effect of data magnitude, described as r×SD.

### 2.2. Fractional Refined Composite Multiscale Fuzzy Entropy

To measure the information inherent in multiscale dataset such as financial and physiology time series, Costa et al. [9] combine the concept of coarsegraining and entropy measure to propose a novel statistic named Multiscale Entropy. Then, FuzzyEn is extended to multiscale case called multiscale FuzzyEn entropy (MFE). Combining fuzzy entropy with multiscale, composite and refine technologies, refined composite multiscale fuzzy entropy (RCMFE) is proposed to detect localized defect of rolling element bearing [28]. The algorithm of RCMFE mainly consists of three procedures. First, for a time series $\left\{x_{i},\;i=1,2,\cdots ,T\right\}$, coarsegraining with scale factor τ is implemented. More precisely, the improved k−th coarse-grained time series $y_{k}^{\left(\tau \right)}=\left\{y_{1,k}^{\left(\tau \right)},y_{2,k}^{\left(\tau \right)},\cdots ,y_{\lfloor \frac{T}{\tau }\rfloor ,k}^{\left(\tau \right)}\right\}$ can be obtained as follows:

(7) $$y_{i,k}^{\left(\tau \right)}=\frac{1}{\tau }\sum_{j=(i-1)\tau +k}^{i\tau +k-1}x_{j}\;1\leq i\leq \left\lfloor \frac{T}{\tau }\right\rfloor ,1\leq k\leq \tau$$

where ⌊u⌋ is the integral part of *u*. Then, for a given scale factor τ, the two defined functions $C_{k,\tau }^{m}\left(r\right)$ and $C_{k,\tau }^{m+1}\left(r\right)$ are calculated for $\{y_{i,k}^{\left(\tau \right)},1\leq i\leq \left\lfloor \frac{T}{\tau }\right\rfloor$} with embedding dimension *m* and m+1. Then, the mean of $C_{k,\tau }^{m}\left(r\right)$ and $C_{k,\tau }^{m+1}\left(r\right)$ for *k* denoted as ${\bar{C}}_{\tau }^{m}\left(r\right)$ and ${\bar{C}}_{\tau }^{m+1}\left(r\right)$ are computed respectively, i.e., ${\bar{C}}_{\tau }^{m}\left(r\right)=\frac{1}{\tau }\sum_{k=1}^{\tau }C_{k,\tau }^{m}\left(r\right)$. Finally, RCMFE can be estimated as

(8) $$\mathrm{RCMFE}\left(x,\tau ,m,n,r\right)=-\ln\frac{{\bar{C}}_{\tau }^{m+1}\left(r\right)}{{\bar{C}}_{\tau }^{m}\left(r\right)}.$$

Obviously, when k=1, the RCMFE degenerates to classic MFE case.

Moreover, a revised entropy measure based on the SampEn and fractal theory in Refs. [35,36], is developed to detect underlying properties of fractional order behavior in a complex system [22]. Inspired by the works [21,22,28], combining RCMFE with fractional order information, a revised complexity measure fractional refined composite multiscale fuzzy entropy (FRCMFE) is proposed to study the complexity behaviors of the returns and volatility of international stock indices in the work.

Then, the corresponding FRCMFE value of a time series {x(t),t=1,2,⋯,T} is calculated as:

(9) $$\mathrm{FRCMFE}\left(x,\tau ,m,n,\alpha ,r\right)=-\frac{\ln{\bar{C}}_{\tau }^{m+1}\left(r\right)-\ln{\bar{C}}_{\tau }^{m}\left(r\right)+\psi \left(1\right)-\psi \left(1-\alpha \right)}{\Gamma (\alpha +1)}{\left[\frac{{\bar{C}}_{\tau }^{m+1}\left(r\right)}{{\bar{C}}_{\tau }^{m}\left(r\right)}\right]}^{-\alpha }$$

where α∈[−1,1] is called fractional order exponent, and when α=0, the FRCMFE degenerates to classic RCMFE case. As a statistic, FRCMFE vitally depends on the choice of parameters *m* and *r*, but there are no guidelines for optimizing them. A widely accepted rule in such kinds of fractional entropy measure by researchers is that r=l×SD(0.1≤l≤0.25) and *m* is 2≤m≤7. We estimate the FRCMFE for all the considered price logarithmic returns and volatility with parameters m=2 and $\hat{r}=0.15\times SD$ in the work, where SD is the standard deviation of coarse-grained time series of the original price returns [22].

## 3. Complexity Measure for Synthetic Data

In the section, we study the complexity behavior of Gaussian white noise (GWN), which is usually applicable to the comparative study with complex models, with different lengths and with MFE, CMFE, and RCMFE measures. We know that the inherent dynamics of Gaussian white noise is invariant, no matter how long series length is. RCMFE can obtain a more stable entropy statistics than MFE and CMFE. The standard deviations of the MFE, CMFE and RCMFE for different data lengths (1000, 1500, 2000, 2500, 3000, 5000, 10000) with two scale factor (τ=10 and τ=20) are list in Table 1. From Table 1, the performance of MFE, CMFE and RCMFE can be evaluated. For all Gaussian white noise, the standard deviations of three kinds of entropy measures decrease with the increase of data length, respectively. Therefore, the accuracy of entropy statistics is affected by the size of data samples. In other words, the longer the length of the time series is, the higher the accuracy of the calculation is. Moreover, compared with CMFE and MFE, the standard deviations of RCMFE in each scale factor are smaller, hence RCMFE can produce the most stable results. It is worth noting that for a fixed data length, standard deviations of entropy measures tends to increase when the scale factor is from 10 convert to 20. This result confirms theoretical analysis of entropy instability, which may be caused by a shorter coarse grain sequence for a bigger scale factor.

**Figure 1:**
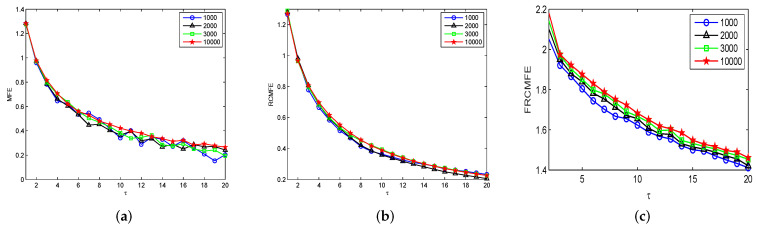
Complexity of Gaussian series with different length: (**a**) MFE, (**b**) RCMFE, (**c**) FRCMFE.

Next, the MFE, RCMFE, and FRCMFE (with α=−0.04) will be comparatively analyzed in terms of their capability to reveal structural differences on GWN series. We further analyze the time series with different numbers of data points with these methods. The MFE, RCMFE, and FRCMFE values of Gaussian series with different scale factor τ are displayed in Figure 1, where scale factor τ is from 1 to 20 with step size 1. In Figure 1, for all series, complexity values decrease with the increase of scale factor τ, fluctuations of the MFE curves are significant larger than those of the RCMFE ones, and the complexity curves with the longest length are the most stable. In addition, MFE and RCMFE cannot discriminate these series significantly, which may cause serious defects in the practical applications. Furthermore, we find that FRCMFE measure obtains larger separation between entropy values in GWN sequences than MFE and RCMFE, hence, FRCMFE can discriminate these signals more significantly than others. To sum up in conclusion, FRCMFE method can effectively overcome the shortcomings of MFE and RCMFE methods, which cannot distinguish significantly GWN series with different length. In addition, FRCMFE method is relatively sensitive and can better discover the inherent properties of time series with different degree complexity.

## 4. Complexity Measure for International Stock Indices

In this section, we explore the complexity behaviors of the daily price returns and volatility of international stock indices. We choose 5 important international stock indices from three countries (i.e., America, Japan and China) to better confirm the application of the introduced method in practical situations. The 5 indices are S&P500 (from America market), N225 (from Japan market), SSE, SZSE, HSI (from China market), respectively. The corresponding dataset is collected from the Yahoo Financial web site (Available online: https://finance.yahoo.com/ (accessed on 8 May 2012)). we select analyzed time interval of returns and volatility is from 30 June 1995 to 31 May 2017, with 4000 data points (but there are some slight differences in time interval because of the slightly different non-trading days in the above stock markets of three countries, and some individual missing data are added by linear interpolation).

### 4.1. Complexity Measure of Returns

**Figure 2:**
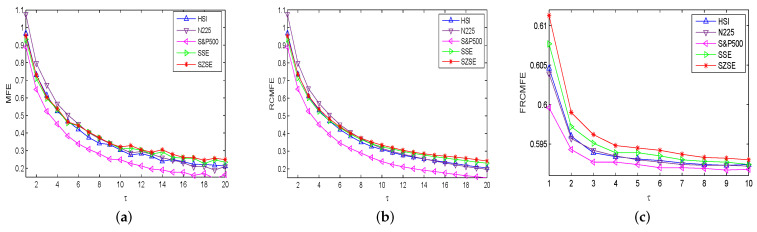
Complexity of returns: (**a**) MFE, (**b**) RCMFE, (**c**) FRCMFE.

The MFE, RCMFE, and FRCMFE analyses are used to survey the complexity dynamics of the price returns {r(t),t=1,2,⋯,4000} of international stock indices. We fix n=2 and r=0.15 in the following for simplification, and the corresponding results are displayed in Table 2, Table 3 and Table 4, and Figure 2. Figure 2 depicts entropy measure values of returns with MFE, RCMFE and FRCMFE methods with scale factor τ from 1 to 20 with step size 1, FRCMFE method with scale factor τ from 1 to 10 with step size 1 (since FRCMFE value changes with scale factor τ from 10 to 20 is very small), where fractional order exponent α is set to be −0.04 in FRCMFE method. In Figure 2, for all price returns, similar to MFE curve, FRCMFE and RCMFE curves decrease with scale τ increase. Moreover, entropy curves of S&P500 and N225 are under those of SSE, SZSE, and HSI in high scale, which may because the America and Japan security markets are more mature and efficient than China security markets, and display more random behavior, while there are long-range correlations in China security markets to some extent. It confirms that the entropy value of series with long-range correlations is theoretically higher than that of a random signal in high scales [9].

Table 2 and Table 3 list MFE and RCMFE values of returns with different time scale factor τ, where we choose scale τ to be 1, 3, 5, 7, 9, 12, 16, 20, respectively. For all series, entropy values decrease with scale increases, and for a fix τ, entropy values of SSE, SZSE and HSI are larger than those of S&P500 and N225, which is similar to Figure 2. Table 4 and Figure 2c display FRCMFE values of returns with different time scale factor τ. Since the FRCMFE value changes mainly focus on the scale factor from 1 to 10 with step size 1, to study more deeply, in Table 4 we take the scale factor τ as 2, 3, 4, 5, 6, 7, 8, 10, respectively. In Figure 2, for MFE and RCMFE methods, the entropy values of Asian market is higher than that of America market, but the Asian market is not well discriminated. However, in the FRCMFE analysis, the difference between the two kinds of markets is more significant to some extent, the separation between entropy curves are larger. This means that FRCMFE can describe the multiscale structure of time series. Meanwhile, we can also see that HSI is closer to the America market than others, which may be because Hong Kong’s financial market still maintains the traditional business mechanism of the British financial market. The entropy values of Hong Kong’s market are slightly higher than those of the America market because its business behavior is also influenced by other Asian markets and some Chinese rules and policies. For example, due to China’s “One Country, Two Systems” policy, the Hong Kong stock market gradually approaches the China market with the increase of the scale factor, and the HSI maintains a good consistency with other Asian capital markets such as Japan. Moreover, by changing the time scale factors τ and fractal exponent α, richer information can be obtained, and the internal dynamics of financial time series can be better detected. Finally, FRCMFE method also clearly distinguishes SSE and SZSE. We get that SZSE has the higher entropy value, maybe it contains more small and medium-sized enterprise (SME) board and growth enterprise markets (GEM) with high activity. We also believe that with the opening of science and technology innovation board (SSE STAR Market), SSE will also possesses a high activity.

**Figure 3:**
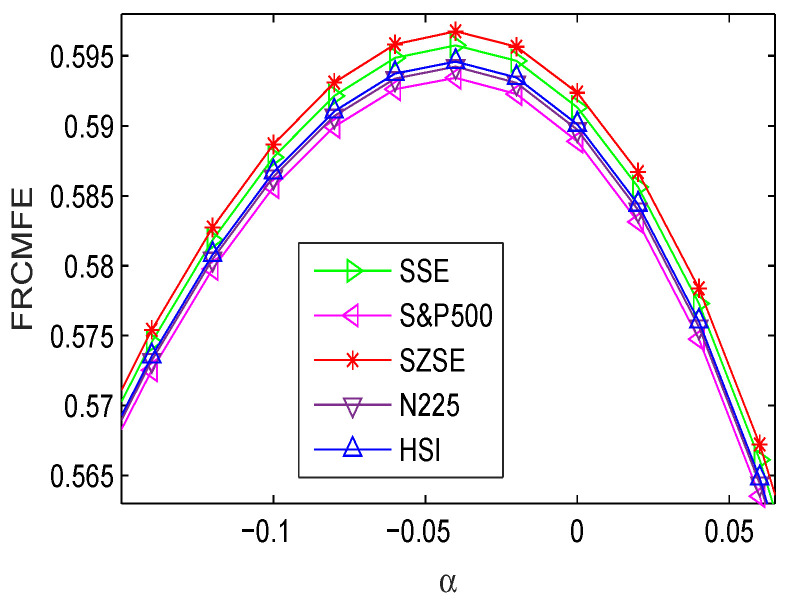
FRCMFE of returns with different α (for τ=5).

Then we use the FRCMFE analysis to explore the complexity dynamics of the returns. Table 5 lists the FRCMFE with different fractional order exponent values α from −0.3 to 0.5 with step size 0.1 and α=−0.04. As exponent α increases, the FRCMFE values increase to the maximum and then decrease quickly. According to Table 5, FRCMFE achieves the maximum value with α at approximately −0.04. Figure 3 depicts FRCMFE (with α=−0.04) curves of return series of different financial indices with different fractional exponent values α, where all curves are significantly separated. This further verifies the feasibility of the FRCMFE method, which can better discriminate different financial time series with different degrees of complexity.

### 4.2. Complexity Measure of Volatility

**Figure 4:**
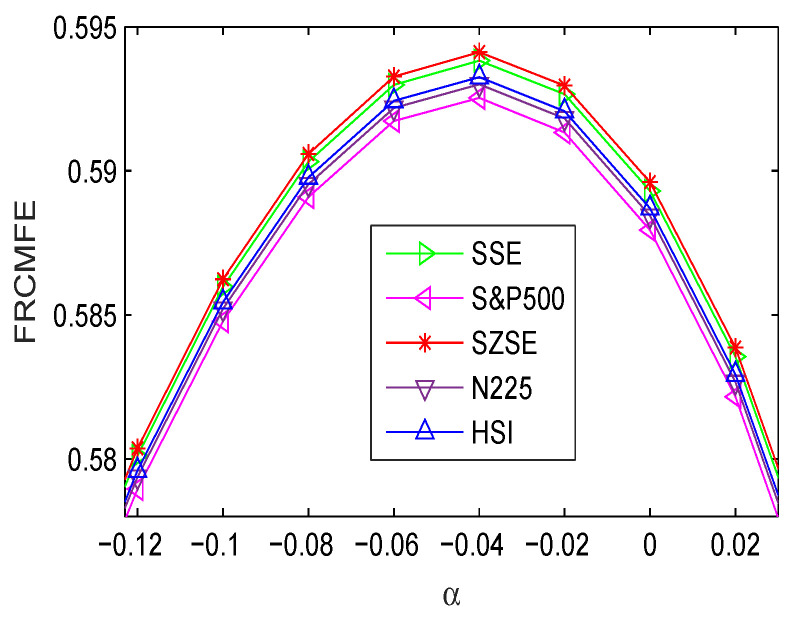
FRCMFE of volatility series with different α (for τ=5).

The MFE, RCMFE, and FRCMFE analyses are used to survey the complexity dynamics of the volatility {|r(t)|,t=1,2,⋯,4000} of international stock indices, and the corresponding results are displayed in Table 6, Table 7, Table 8 and Table 9, and Figure 4. Table 6 lists the FRCMFE of {|r(t)|} with different fractional exponent values α, where α ranges from −0.3 to 0.5 with step size of 0.1 and α=−0.04, the FRCMFE values first increases and get the maximum around α=−0.04, and then decreases.

Table 7 and Table 8 list MFE and RCMFE of {|r(t)|} with different time scale factor τ=1,3,5,7,9,12,16,20, similar to Table 2 and Table 3, entropy values decrease with scale increases, and for a fix τ, almost all entropy values of SSE, SZSE and HSI are larger than those of S&P500 and N225. It is interesting that entropy values of all volatility series are smaller than those of returns in low scale and larger than those of returns in high scale for a fix τ, which confirms that the entropy value of series with long-range correlations is theoretically higher than that of a random signal [9], since volatility clustering reveals that absolute return series exhibit significant autocorrelation.

Table 9 lists FRCMFE of {|r(t)|} with different time scale factor τ=2,3,4,5,6,7,8,10, (since the FRCMFE value changes are not significantly on the scale factor from 10 to 20 with step size 1.) similar to Table 7 and Table 8, entropy values decrease with scale increases, and for a fix τ, almost all entropy values of SSE, SZSE and HSI are larger than that of S&P500.

**Figure 5:**
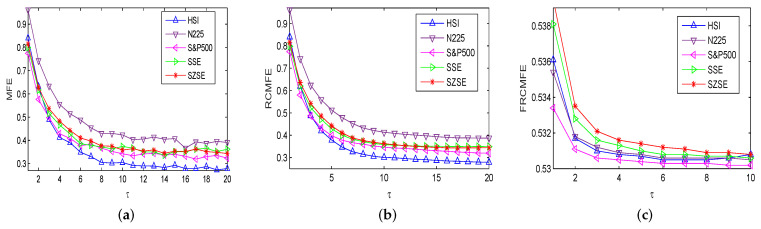
Complexity of volatility series: (**a**) MFE, (**b**) RCMFE, (**c**) FRCMFE.

Figure 4 depicts FRCMFE curves of volatility series with different fractional exponent values α. Compared with Figure 3, Figure 4 has the similar dynamics behaviors. Moreover, the FRCMFE of volatility series are significantly decrease, which means that the volatility series exhibit lower complexity than the return series. Figure 5 depicts the complexity of financial volatility time series with MFE, RCMFE, and FRCMFE with scale factor τ from 1 to 20 with step size 1, where fractional order exponent α is set to be −0.04. Similar to Figure 2, for all price returns, MFE, FRCMFE, and RCMFE curves decrease with scale τ increase.

## 5. Conclusions

In the work, a novel complexity measure, i.e., FRCMFE, is presented by combining RCMFE method with fractal theory, which can stably evaluate structural dynamics and detect underlying fractional order behavior in a complex system. Then, we survey the complexity behavior of GWN series with different lengths with MFE, CMFE, RCMFE and FRCMFE measures, which shows that RCMFE and FRCMFE is more stable and sensitive than traditional methods (showing larger separation between entropy values of financial series than traditional measures), and they are suited to analyze short-term financial time series with noise. Next, we investigate the complexity behavior of price logarithmic returns and volatility of international stock indices. The results show that entropy values of all volatility series are smaller than those of returns in low scale and larger than those of returns in high scale for a fix τ, which coincides with previous literature of multiscale entropy. Moreover, FRCMFE can distinguish different financial markets sensitively and significantly.

## Figures and Tables

**Table 1:** Standard deviations of MFE, CMFE and RCMFE of Gaussian white noise.

Method	Data Length						
	1000	1500	2000	2500	3000	5000	10,000
MFE (τ = 10)	0.0827	0.0794	0.0672	0.0483	0.0470	0.0339	0.0302
CMFE (τ = 10)	0.0706	0.0538	0.0449	0.0378	0.0331	0.0313	0.0185
RCMFE (τ = 10)	0.0684	0.0509	0.0466	0.0366	0.0327	0.0283	0.0180
MFE (τ = 20)	0.0885	0.0823	0.0788	0.0663	0.0717	0.0498	0.0337
CMFE (τ = 20)	0.0763	0.0681	0.0568	0.0544	0.0397	0.0340	0.0290
RCMFE (τ = 20)	0.0716	0.0658	0.0517	0.0397	0.0393	0.0337	0.0228

**Table 2:** MFE of returns with different τ.

τ	SSE	S&P500	SZSE	N225	HSI
1	0.9329	0.8888	0.9533	1.0754	0.9673
3	0.5968	0.5235	0.6029	0.6736	0.6139
5	0.4574	0.3833	0.4637	0.5034	0.4672
7	0.4049	0.3077	0.4059	0.4016	0.3750
9	0.3427	0.2518	0.3411	0.3417	0.3352
12	0.3032	0.2127	0.3050	0.2948	0.2836
16	0.2576	0.1771	0.2621	0.2317	0.2371
20	0.2346	0.1635	0.2493	0.2099	0.2131

**Table 3:** RCMFE of returns with different τ.

τ	SSE	S&P500	SZSE	N225	HSI
1	0.9329	0.8888	0.9533	1.0754	0.9673
3	0.6022	0.5263	0.6134	0.6550	0.6108
5	0.4715	0.3948	0.4824	0.5058	0.4709
7	0.3965	0.3154	0.4017	0.4070	0.3846
9	0.3414	0.2637	0.3517	0.3418	0.3272
12	0.2984	0.2116	0.3053	0.2826	0.2773
16	0.2615	0.1765	0.2722	0.2332	0.2381
20	0.2319	0.1492	0.2442	0.1975	0.2062

**Table 4:** FRCMFE of returns with different τ.

τ	SZSE	SSE	HSI	N225	S&P500
2	0.5990	0.5972	0.5960	0.5957	0.5943
3	0.5962	0.5951	0.5939	0.5942	0.5927
4	0.5948	0.5939	0.5934	0.5935	0.5927
5	0.5945	0.5939	0.5931	0.5930	0.5924
6	0.5942	0.5935	0.5929	0.5927	0.5920
7	0.5937	0.5930	0.5926	0.5924	0.5920
8	0.5933	0.5928	0.5924	0.5923	0.5919
10	0.5930	0.5924	0.5924	0.5922	0.5918

**Table 5:** FRCMFE of returns with different α.

α	SSE	S&P500	SZSE	N225	HSI
−0.3	0.4811	0.4795	0.4817	0.4801	0.4803
−0.2	0.5457	0.5439	0.5465	0.5445	0.5448
−0.1	0.5877	0.5856	0.5887	0.5863	0.5867
−0.04	0.5958	0.5934	0.5967	0.5942	0.5946
0	0.5913	0.5889	0.5924	0.5897	0.5901
0.1	0.5341	0.5314	0.5353	0.5323	0.5328
0.2	0.3822	0.3794	0.3854	0.3803	0.3808
0.3	0.0794	0.0767	0.0806	0.0776	0.0780
0.4	−0.4777	−0.4800	−0.4767	−0.4792	−0.4788
0.5	−1.5028	−1.5038	−1.5024	−1.5034	−1.5033

**Table 6:** FRCMFE of |r(t)| with different α.

α	SSE	S&P500	SZSE	N225	HSI
−0.3	0.4798	0.4789	0.4800	0.4793	0.4794
−0.2	0.5442	0.5432	0.5444	0.5435	0.5437
−0.1	0.5860	0.5848	0.5862	0.5852	0.5854
−0.04	0.5938	0.5929	0.5941	0.5930	0.5933
0	0.5893	0.5879	0.5896	0.5884	0.5887
0.1	0.5319	0.5304	0.5322	0.5309	0.5312
0.2	0.3799	0.3783	0.3802	0.3788	0.3792
0.3	0.0771	0.0756	0.0775	0.0761	0.0764
0.4	−0.4796	−0.4809	−0.4793	−0.4804	−0.4802
0.5	−1.5036	−1.5041	−1.5035	−1.5039	−1.5038

**Table 7:** MFE of |r(t)| with different τ.

τ	SSE	S&P500	SZSE	N225	HSI
1	0.9614	0.7745	0.8403	0.7986	0.8148
3	0.6337	0.4977	0.4889	0.5158	0.5367
5	0.5148	0.4080	0.3897	0.4275	0.4427
7	0.4538	0.3826	0.3299	0.3783	0.3967
9	0.4301	0.3523	0.3021	0.3633	0.3747
12	0.4057	0.3409	0.2895	0.3496	0.3539
16	0.3661	0.3314	0.22782	0.3505	0.3519
20	0.3909	0.3230	0.2785	0.3615	0.3433

**Table 8:** RCMFE of |r(t)| with different τ.

τ	SSE	S&P500	SZSE	N225	HSI
1	0.7986	0.7745	0.8148	0.9614	0.8403
3	0.5236	0.4868	0.5245	0.6238	0.4876
5	0.4284	0.3985	0.4418	0.5123	0.3783
7	0.3821	0.3668	0.3876	0.4522	0.3261
9	0.3631	0.3519	0.3684	0.3684	0.3056
12	0.3537	0.3410	0.3535	0.4037	0.2954
16	0.3494	0.3280	0.3437	0.3904	0.2851
20	0.3491	0.3197	0.3440	0.3868	0.2791

**Table 9:** FRCMFE of |r(t)| with different τ.

τ	SSE	S&P500	SZSE	N225	HSI
2	0.5328	0.5311	0.5335	0.5318	0.5317
3	0.5316	0.5306	0.5321	0.5312	0.5310
4	0.5313	0.5305	0.5316	0.5309	0.5308
5	0.5310	0.5304	0.5314	0.5308	0.5307
6	0.5308	0.5303	0.5312	0.5306	0.5305
7	0.5308	0.5303	0.5311	0.5306	0.5305
8	0.5307	0.5303	0.5309	0.5306	0.5305
10	0.5306	0.5302	0.5308	0.5305	0.5308

## Abbreviations

The following abbreviations are used in this manuscript:

FRCMFE	Fractional refined composite multiscale fuzzy entropy
FuzzyEn	Fuzzy entropy
CMFE	Composite multiscale fuzzy entropy
RCMFE	Refined composite multiscale fuzzy entropy
MFE	Multiscale fuzzy entropy
