# Erratum: Wu, Z., Zhang, W. Fractional Refined Composite Multiscale Fuzzy Entropy of International Stock Indices. *Entropy* 2019, 21(9), 914

**DOI:** 10.3390/e22070777

**Published:** 2020-07-16

**Authors:** Zhiyong Wu, Wei Zhang

**Affiliations:** School of Mathematics and Physics, and the Key Laboratory of Advanced Perception and Intelligent Control of High-End Equipment, Ministry of Education, Anhui Polytechnic University, Wuhu 241000, China; wuzhiyong10@gmail.com

We have found an error in grant number in Funding published in Entropy [1]:

       KJ2019A014

that should be corrected to the following in this paper [1]:

       KJ2019A0141

The authors would like to apologize for any inconvenience caused to the readers by these changes.
